# Psychosocial impact of scars due to cutaneous leishmaniasis on high school students in Errachidia province, Morocco

**DOI:** 10.1186/s40249-017-0267-5

**Published:** 2017-04-07

**Authors:** Issam Bennis, Séverine Thys, Hind Filali, Vincent De Brouwere, Hamid Sahibi, Marleen Boelaert

**Affiliations:** 1 National School of Public Health, Ministry of Health, Rabat, Morocco; 2 Department of Public Health, Institute of Tropical Medicine, Antwerp, Belgium; 3 Department of Biomedical Sciences, Faculty of Pharmaceutical, Biomedical and Veterinary Sciences, University of Antwerp, Antwerp, Belgium; 4 Hassan II Institute of Agronomy and Veterinary, Rabat, Morocco; 5 Ecole Nationale de Santé Publique, Rue Lemfedel Cherkaoui, Madinat Al Irfane, 10000 Rabat Morocco

**Keywords:** Belief, Body image, Cutaneous leishmaniasis, *Leishmania major*, Scars, Self-concept, Self-stigma, Social stigma, Students, Adolescents, Errachidia, Morocco

## Abstract

**Background:**

In Morocco, cutaneous leishmaniasis (CL) is usually known to be a slowly healing localized skin disease, but in some cases, it can lead to mutilating scars. The outbreak of CL due to *Leishmania major* in the Errachidia province in southeastern Morocco between 2008 and 2010 left many adolescents with permanent scar tissue on the face or other exposed body parts. We studied the psychosocial impact of CL on these young people.

**Methods:**

In 2015 we conducted a cross-sectional survey among high-school students living in boarding schools in two CL-endemic areas of Errachidia: Rissani and Tinejdad. A self-administered questionnaire elicited responses about general knowledge of CL and related scars. An open-ended question focused on the possible psychosocial effects associated with these scars. The quantitative data were analyzed with Epi Info™ and the text data with NVivo software.

**Results:**

Almost 20% of 448 respondents reported they had experienced a CL lesion and 87% said it could possibly or definitely lead to psychological consequences. The text analysis showed that girls more often than boys expanded on the negative psychological effects of CL. The students considered CL as “dangerous”, “serious”, and “deathly”, and said it sometimes led to extreme suicidal ideations.

**Conclusions:**

The burden of CL in this age group is not negligible. The indelible CL scars lead to self-stigma and social stigma, and the emergence of negative psychological effects in this age group. While some students accepted their CL scars and related suffering as their “destiny”, others were eagerly demanding protective measures against CL and treatment for the scars.

**Electronic supplementary material:**

The online version of this article (doi:10.1186/s40249-017-0267-5) contains supplementary material, which is available to authorized users.

## Multilingual abstracts

Please see Additional file 1 for translations of the abstract into the five official working languages of the United Nations.

## Background

Leishmaniasis, a parasitic disease of the genus *Leishmania* transmitted by a sand-fly vector, is one of the most neglected diseases in the world, affecting the poorest of the poor [1]. In low- and middle-income countries, the affected population lives in precarious dwellings within vulnerable environmental conditions [2].

Cutaneous leishmaniasis (CL), the most frequent clinical presentation, usually presents as a localized lesion at the site of the sand-fly bite after a lapse of several days to months [2]. The lesion starts as an erythema that gradually transforms to a papule and later to a nodule. The nodule then increases in volume and progressively ulcerates [3]. The whole process generally takes between two and six months [4]. In immunocompetent persons, CL lesions are slowly self-healing but they often lead to scar tissue [5]. As the sand flies bite most often in the face or other exposed parts of the body, these disfiguring scars can lead to substantial psychological and social suffering, and economic losses [6]. Lesions affecting the central area of the face have a higher impact compared with others [7].

Studies conducted in Afghanistan, Pakistan, Syria, and Iran have demonstrated the serious social consequences of CL for young women. They may not be able to get married in the future [8–11] or not be allowed to stay with their partner if they contract CL after marriage [12]. Often, persons affected by CL during childhood only become aware of their affected body image during adolescence when the indelible scars become more visible in their own eyes and/or the eyes of their loved ones [13]. Indeed, perception of the body, more precisely the self-perceived beauty of the face, is a major factor influencing self-awareness, especially, but not only, in women. In contrast, a similar facial scar in a male subject could be considered attractive in some communities [14].

Interestingly, a recent study quantified the CL burden by estimating the related disability-adjusted life years lost (DALY) based on physical disfigurement only. The authors of that study stated that the CL burden calculated in this way did not differ significantly in terms of age in the same geographic region, probably because this approach does not take into account the social stigma, or the emotional or financial impact of CL [15]. However, as also demonstrated in the case of lymphatic filariasis, the disabling or disfiguring sequelae of some neglected tropical diseases can have a substantial impact on mental health [16].

In Morocco, CL is caused by two species, *L. major* and *L. tropica.* The first is a zoonotic disease with rodents as a reservoir host, the second is anthroponotic [17]. CL due to *L. major* has recently led to important epidemics. The Errachidia province in southeastern Morocco has seen an epidemic peak in most of its districts between 2008 and 2010 [18, 19]. The most affected age group was those aged between 11 and 20 years, which is rarely studied in literature.

Therefore, the aim of this article is to describe the psychosocial impact of CL on adolescents in Morocco’s *L. major*-endemic areas.

## Methods

### Conceptual framework

**Fig. 1:**
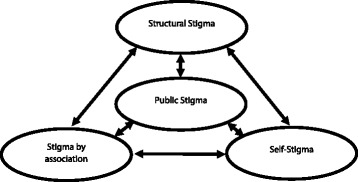
Conceptualization of ‘stigma’, based on the framework developed by Bos et al. [20] and Pryor et al. [21]

Our concept of stigma is rooted in the framework proposed by Bos et al. [20], which was adapted from the one developed by Pryor et al. [21] (see Fig. 1). Stigma is categorized by these authors as belonging to one of four types. The public (or social) stigma is at the core of the model and refers to the social and psychological reactions of society to the person who has the stigmatized condition [22]. Self-stigma reflects the impact of stigma on the stigmatized person and is partly internalized through a reduction in self-worth and psychological distress. Stigma by association reflects the negative reactions to family and friends of stigmatized persons and their attitudes to this [23]. Then, once the stigma becomes institutionalized within society, the authors define the fourth category as “structural stigma”, when the ideological systems of society perpetuate the stigmatized status [21].

As stated above, those who internalize stigma often experience significant loss of self-esteem [22]. The relationship between self-concept and self-stigma is well documented in psychiatry. Stigmatized people have negative attitudes towards themselves as a reaction to their condition [24]. Self-concept should be distinguished from self-awareness and self-esteem. McConnell et al. [25] state that the awareness about the self is always influenced by the context. Over time, the person develops a self-concept, which interacts with self-esteem, self-knowledge, and self-awareness within the social context. In this article, we use the term self-concept as the cognitive description of one’s self (self-awareness) added to the opinion about one’s self (self-esteem).

### Study population

In April 2015, we carried out a cross-sectional questionnaire survey among boarding school students in two areas of the Errachidia province that reported high CL attack rates. The choice of these two areas was based on the reported incidence of CL cases, as published earlier [18]. One school was located in Tinejdad city with boarding facilities serving the surrounding districts of the Tinejdad Ferkla area. The second school was located in Rissani city serving the districts of the Rissani Sfalate area (see Table 1).

**Table 1:** Origin of interviewed boarding school students, stratified by gender (*n* = 448)

	District	Gender		Total
		Girls	Boys	
Tinejdad Area	Tinejdad	06	03	09
	Ferkla Oulia	10	10	20
	Ferkla Soufla	35	28	63
	Aghbalou	69	113	182
	Others	00	03	03
	Sub-total	120	157	277
Rissani Area	Rissani	10	08	18
	Sidi Ali	00	11	11
	Sfalate	18	01	19
	Taous	42	80	122
	Others	00	01	01
	Sub-total	70	101	171
	Total	190	258	448

The two schools enrolled a total of 3 246 students including 561 boarding students with separate facilities for the girls and the boys. Henceforward, we will use the codes “FT” or “MT” to identify respectively the group of girls (F) and boys (M) from the Tinejdad (T) area, and the codes “FR” and “MR” to identify the respective groups of girls and boys from the Rissani (R) area.

### Survey questionnaire

The survey was based on a self-administered questionnaire developed to assess the students’ general knowledge about CL. We pre-tested the questionnaire on 10 students from Errachidia city and found that the time it took to fill out the form was about 10 min. In each high school, the questionnaires were distributed on the same day to all present boarding school students. The questionnaire was in Arabic (English translation in Additional file 2, original Arabic form in Additional file 3).

The introductory part consisted of an information sheet and consent section for voluntary participation in the study, with some administrative information. The questionnaire itself consisted of 18 closed-ended questions and concluded with one open-ended question about the perceived psychological effects of CL scars (see Additional file 4).

As a start, the principal investigator (the first author) explained the purpose of the study and the confidentiality conditions to the students during an extracurricular study session. It was also stressed that the students were free to refuse to partly or completely answer the questionnaire. Then, he invited the respondents to select their answers and write a short paragraph for the last question. Students did not communicate with each other while answering the questions and a boarding master supervised each group. At the end, students returned the questionnaires to the investigator one by one (see Additional file 5 for all student responses).

### Data analysis

Regarding the 18 closed-ended questions of the survey, the data entry, initial data cleaning, and descriptive analysis were done using Epi Info™ 7 (CDC Atlanta, USA) software. Three socio-epidemiological attributes were chosen for the comparative analysis: 1) participant’s gender: female vs. male; 2) a history of being affected by CL (personal experience with CL): yes or no; and 3) area of residence: Tinejdad or Rissani.

**Fig. 2:**
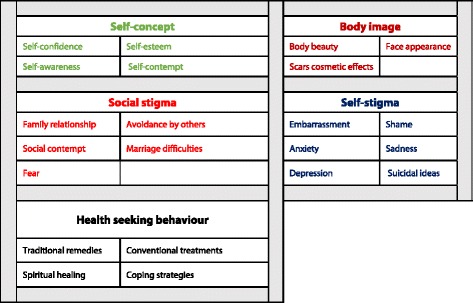
Nodes and codes used in the text analysis (with NVivo 10 software)

Students’ answers to the open-ended questions required the verbatim transcription in the Arabic language and then translation into French. The text analysis was performed with NVivo software version 10 (QSR International Melbourne Australia). The coding followed a deductive approach based on the conceptual framework (see Fig. 1). An inductive analysis was done to extend the generated codes (see Fig. 2) in order to define the factors which influenced the impact of CL scars on the patient’s psychological state. Five major themes were elucidated: perception of body image, self-stigma, social stigma, self-concept, and health-seeking behavior. Attributes of gender, area of residence, and personal CL experience were also used to classify answers in order to examine relationships within these qualitative data.

## Results

From the 454 students present on the day of the survey, 258 boys and 190 girls (448 in total) participated in the study, while six boys refused. The mean age of the respondents was 18.1 years ± 2.3 for boys and 17.2 years ± 1.6 for girls. Table 1 shows the origin of the students stratified by gender. All participants were from rural districts that declared CL outbreaks between 2008 and 2010 [18].

### Affliction with and general knowledge about CL

In total, 362 out of 448 respondents (80.8%) said they knew about CL and 60% correctly indicated that an insect transmits CL to humans. Eighty-eight participants (19.6%) reported they personally suffered from CL (38 girls, 50 boys) and among the others, 159 knew at least one person who was affected by CL. In the group of 88 students who developed CL, 71 remembered the specific year that they were affected. Most cases appeared between 2008 and 2010.

When asked if they knew CL by any local name, 181 participants out of 448 replied “*Leishmania*”. Twenty wrote a name connected with a mosquito and 15 described CL as a boil or a sore such as the “boil of Ata”. (The Ata is an ethnic group living outside the Errachidia province). Finally, two respondents noted “azmoulen”, an Amazigh word for “scar”. A minority of 26 students wrote the name of a non-related skin disease, such as measles, eczema, syphilis, jaundice, or urticaria.

The majority of respondents (*n* = 356) stated that CL leaves marks and scars on the skin, usually on the face (*n* = 315). Girls more often than boys pointed out that CL leaves scars and that these scars are on the face.

Half of the students evoked the existence of medical treatment against CL scars, while one third quoted traditional remedies such as henna, eucalyptus, saffron, aloe vera, tea, *Artemisia*, Sahara grass, among others. Some mentioned food items such as olive oil, lemon, eggs, honey, onion, and garlic. Others evoked traditional practices such as burning the diseased skin, the use of tar, acids (bleach, alcohol, water mixed with bleach powder), or urine of animals. In addition, four students referred to the use of expired drugs (pills blended together or the application in powder form of an outdated injectable antibiotic) in order to treat CL lesions. However, no one reported any specific traditional remedy against CL scars.

Almost all respondents (384/448 or 86%) wrote that CL possibly or definitely has psychological effects. Table 2 shows that there was no significant difference in response to this question related to gender or personal experience with CL, although almost twice as many students from the Rissani area (12.8%) considered there were no psychological consequences than students from the Tinejdad area (6.6%).

**Table 2:** Potential psychological effects of CL scars on students, stratified by gender, area of origin, and personal CL experience (*n* = 422)

Attributes		Psychological effects of CL scars^a^			
		Yes	May be	No	
Gender	Girls (*n* = 178)	82	81	15	
	Boys (*n* = 244)	99	122	23	*P* = 0.53
Origin	Rissani area (n = 164)	74	69	21	
	Tinejdad area (*n* = 258)	107	134	17	*P* = 0.03
Personal CL experience	Personal CL experience (*n* = 86)	42	35	09	
	No Personal CL experience (*n* = 333)	137^b^	167^c^	29	*P* = 0.29

^a^26 missing data

^b^Two non-respondents to this question

^c^One non-respondent to this question

The text analysis of the open-ended question about the possible CL impact is presented below in three thematic sections: 1) CL severity and body image; 2) CL scars and stigma; and 3) dealing with scars. Twenty percent of the boys (52/258) and thirteen percent of the girls (24/190) did not answer the open-ended question.

### CL severity and body image

The students’ perception of the severity of CL can be summarized in three interrelated words: “dangerous”, “serious”, and “deathly”.

#### Dangerous

CL is perceived by many as dangerous because of the fear of contagion and the risk of transmitting CL to relatives: *“It will lead to social isolation and the person will live away from others due to fear of contaminating them.”* (MT305). This fear includes the fear of being rejected by their own relatives: *“The person affected is afraid from this disease as there is a risk it could worsen and not heal. He will be disturbed by its appearance if seen [by others] and be afraid that his friends and family will become distant from him and reject him for fear of being affected by the same disease.”* (MR081). Another boy wrote: *“He will not be able to share meals with his family.”* (MT360).

#### Serious

One girl wrote: *“The affected person will be disgusted to see himself in the mirror.”* (FT186). Another wrote: *“Sometimes the affected people end up hating themselves.”* (FR044). Differences according to gender were observed in terms of use of the word “scar”. Many girls but very few boys thought that CL scars are considerably worse for a woman than for a man. According to our respondents, girls are more concerned about the effects of CL scars, the appearance of their face, and the beauty of their body. Three boys reported that men are more heavily affected by CL, as they lose their masculinity as a result of the disease. One boy evoked the need for psychological support: *“… Also it causes the patient psychological and dermatological effects over a long period of time, which requires a visit to a psychiatrist.”* (MR121). One girl noted that both sexes suffered equally due to the disease (FR031).

#### Deathly

A few respondents thought (erroneously) that CL can have a fatal outcome and wrote that death is the natural outcome of the disease. Interestingly, others evoked a fatal outcome in the context of suicidal thoughts, as stated by one boy: *“This disease often influences some people because it leaves scars from the beginning that lead a person to commit suicide.”* (MR123). In addition, four girls cited the terms “depression” and “suicidal ideations” in their answers. One girl noted: *“Cutaneous leishmaniasis influences the psychological state and leads to death.”* (FT289).

### CL scars and stigma

The fear of facing others was frequently mentioned by both sexes. *“…The affected person cannot talk about his disease due to fear of being rejected by people,”* noted one boy (MR132). *“The person affected cannot show this to his friends because they will not want to sit with him,”* said another boy (MR160). The attitude of friends and family influences the psychological state of the affected person, as reported by one girl: *“She will have depression, a durable fear, and shame. She does not have the absolute courage to sit with her friends for fear of their mockery.”* (FT237).

Additionally, girls noted components of self-stigma such as shame, embarrassment, depression, and self-contempt. One girl noted: *“The affected person is very worried…. and feels ashamed in the presence of people. She prohibits herself to go to rallies because of this disease.”* (FT 225). Another wrote: *“The sequelae of leishmaniasis negatively influence the condition of the affected person. A psychological complex will develop and she will be ashamed to appear in front of friends because it is a mark of shame and contempt.”* (FT265).

The answers show how self-stigma and social stigma are linked. When the scar is formed, people tend to stare at it and the gaze of others induces social rejection of the affected person. *“It affects me every time I meet my friends. They look at my scar, it diminishes my value in front of people,”* reported a girl who experienced CL (FR009). Consequently, communication barriers emerge: *“The scar is a mark of shame and contempt. The affected person will be unable to cope with society due to fear of social discrimination and contempt.”* (MT413). One girl noted: *“She will become closed off (introverted) and she will not talk to people and she is not going to love looking at herself because of these scars.”* (FT243). The affected person no longer has the same appearance as before, and she/he is now different from others. The negative behavior of others leads to a feeling of isolation and difficulty in everyday life: *“There is a feeling of isolation and loneliness, and a lack of stability in daily life because of the negative attitude of society towards the patient,”* wrote one boy (MR120). Another girl wrote: *“For a girl when the disease leaves a mark on the face for example, the girl will think that is dangerous for her beauty, which will influence her psychological state. Especially in our traditional society that is absolutely not lenient towards those who have spots on the face because they think it is hereditary.”* (FT194). CL can lead to ‘social death’ as explained by one girl: *“Moroccan society is not merciful and judges people’s appearance.”* (FT241). Indeed, some participants linked CL scars with diminished chances of getting married: *“Often the scars in affected men and especially in affected women are a barrier to getting married because the scars are visible, something that is not tolerated by fiancés.”* (FR049). Another girl noted: *“Women have more fear on their faces, afraid that the young man who comes forward to ask for her hand will disappear after having seen these stains.”* (FR001). A girl who previously experienced CL noted that the possibility of not getting married is a problem only for women: *“She will be afraid about her future especially for the wedding. Meanwhile, in our society, an affected boy remains a man. There is no harm if he has scars.”* (FT202).

In addition, the fear to meet others leads to a lower self-concept: *“The girl is ashamed to show her face; scars can prevent her to leave the house, thereby increasing her psychological suffering”* (FR018). “Why me?” is a question that a person affected by CL often asks: *“Why am I the person who has this disease and carries this mark on the face?”* reported a boy previously affected by CL (MR153)*.*

### Dealing with scars

Half of the respondents wrote that treatment for CL scars was available in hospitals from general practitioners or dermatologists. However, the affected person slowly understands after trying various treatments that these scars will never disappear: *“The psychological state of the affected person can worsen after receiving treatment because the problem is that scars never disappear [even after treatment],”* noted a boy previously affected by CL (MR116). *“The fear and worry regarding the lack of treatment for this disease is the real problem for a person affected by it,”* added a girl also previously affected by CL (FR011). The ineffectiveness of delayed treatment was also something stressed upon: *“We need prompt treatment for this disease to avoid any effect on the psychological state of the person affected.”* (MR174). In addition, one girl mentioned the problem of unaffordable treatment (FT224). Nevertheless, a number of responses included questions about what was indeed an effective treatment against CL scars. *“Is there a way to heal the scars?*” wrote one girl (FR 068). Another girl wrote: *“Is there a treatment for scars? I wish there was a yes answer, there was a cure!”* (FT195).

Various coping strategies were described as a solution to deal with the scars, especially among girls, focusing on ways to hide the scars temporarily: *“I am personally affected by this disease. I suffer from its consequences. The scar on my face has created a big problem in my life. I am obliged to put cream to try to hide it before going anywhere.”* (FR035).

Alternatively, spiritual factors such as ‘God’s will’ and ‘destiny’ were the third care-seeking explanations. Two boys and one girl mentioned that God decided who is to be affected by the disease and who will be healed. One boy also suggested these scars need to be accepted *“…After a while, he will get used to those scars and it will become normal.”* (MR096). Contrastingly, other students asked for direct government intervention: *“This disease could leave psychological trouble in the affected person. That is why the government and the concerned commission must find a solution for this disease, it’s not the duty of the population,”* wrote one boy (MR093). Another boy noted that the population along with the policymakers should be involved in the prevention of this dangerous disease (MT355).

## Discussion

This study showed that 20% of the boarding school students who were surveyed in this CL-endemic region of Morocco had been affected by CL in the past and almost all believed there were potentially psychological implications. CL was found to be relatively well known. Our findings show that CL and its ensuing scars led to a considerable psychosocial burden in this adolescent age group. Girls seemed to be more affected than boys, but both genders equally expressed their concerns and demands for treatment. In this context, adequate dermatological care was beyond the reach of most of the participants due to the poverty of their families. It should be noted that in this Moroccan sociocultural context, scarification marks are common in rural and remote areas in the elderly population and people also contract scars frequently in agricultural work. Traditional tattoos are considered a sign of beauty, a mark of tribal identification, or a protection against sin and malicious witchcrafts [26]. However, nowadays this cultural practice is less commonly accepted by the younger generation living in the same area, and none of the adolescents referred to it. A large majority of them highlighted the negative psychological consequences and the stigmatizing effect of CL and its scars.

Students pointed to a large spectrum of negative psychosocial effects, ranging from slight shame to suicidal thoughts. Social exclusion of CL patients has also been reported elsewhere in literature [8]. In its most extreme form, students equated CL with ‘social death’ due to the visibility of CL scars, which has also been reported by other authors [27–29]. If a CL scar is located on the face, it is perceived as a barrier for marriage and in this traditional society, this can imply ‘social death’ for women. The participants in this study made comments to this effect spontaneously, without any trigger related to marriage built into the questionnaire. This therefore reflected a deep concern in the adolescent age group. The finding is consistent with previous research reporting that CL scars had a deeper impact for women who remained unmarried, especially in a society where early marriages for girls are common [12].

In addition, while several respondents viewed CL scars as a curse, others accepted them within a spiritual context, which requires praying, asking for forgiveness, and having patience and faith that God has a treatment for every disease or a plan for it whether it will be treated or not.

The findings in this study illustrate the conceptual model of stigma developed by Pryor and Bos and colleagues (see Fig. 1). In this traditional Moroccan society, the presence of skin lesions on the face and other exposed body parts leads to public (social) stigma, at least in the adolescent age group, as the cognitive, affective, and behavioral reactions of people to a skin lesion affecting the face are negative and lead to social avoidance [30]. Notions of contagion, of congenitally transmitted infections, and of fatal illness are associated with lesions. In this context, the presence of a CL scar leads to a depreciation of body image in the affected person, which is of course dependent on age and gender, but female adolescents are highly vulnerable, as shown in the literature [31, 32]. The ensuing self-devaluation [28, 33] and deterioration of self-concept [23] leads to self-stigma that is perpetuated due to the protracted nature of the scars. Hence, the explicit demand for this age group is to treat CL more quickly and effectively.

Our study had some limitations. Firstly, the self-administered questionnaire method inevitably provides less detailed information than an individual in-depth interview would generate. For instance, we do not have in-depth explanations for the underlying reasons of shame and contempt induced by CL, and we see a need to conduct focus group discussions in the future to better document the social representations of this disease. However, we chose the method of using a self-administered questionnaire format for ethical reasons, as it would have been difficult to arrange individual interviews in an anonymous way in this environment of a boarding school. We wanted to avoid enhancing harassment and mockery between students in such a closed environment. Secondly, there was a relatively large group of non-respondents to the open-ended question (*n* = 76). This could be explained for 59 participants by the fact that they did not know about the disease or any person affected by it. Thirdly, the previous personal CL scar experience was based only on the self-declaration of the participants and we had no formal verification of this information.

## Conclusions

This is the first study done in Morocco documenting the psychological effects of CL scars in the adolescent population living in CL-endemic areas. The indelible CL scars lead to self-stigma and social stigma, and the emergence of negative psychological effects in this age group. Preventing the avoidable burden of CL and mitigating its dermatological and psychosocial consequences should be a priority for health authorities. We suggest that the control of CL in the North African region should be envisaged in an interdisciplinary, multi-sectorial approach, preferably in a regional framework to prevent as much as possible the avoidable suffering.

## Additional files

Additional file 1: Multilingual abstracts in the five official working languages of the United Nations. (PDF 763 kb)

Additional file 2: English translation of the self-administered questionnaire. (PDF 327 kb)

Additional file 3: Original version of the questionnaire in Arabic. (PDF 1639 kb)

Additional file 4: French translation of participants’ responses to the last question in the questionnaire: Could you write a small paragraph about the probable psychological state of the person (woman or man) affected by those scars? (PDF 404 kb)

Additional file 5: Access database file including full questionnaire and student responses. (ACCDB 1416 kb)

## Abbreviations

CL: Cutaneous leishmaniasis
FR: Female from Rissani area
FT: Female from Tinejdad area
MR: Male from Rissani area
MT: Male from Tinejdad area
