# Supplementary material for: Psychosocial impact of scars due to cutaneous leishmaniasis on high school students in Errachidia province, Morocco
Source: Infect Dis Poverty. 2017 Apr 7;6:46. doi: 10.1186/s40249-017-0267-5 (PMC5383955; doi:10.1186/s40249-017-0267-5)
Supplement: Supplementary file 1 — Multilingual abstracts in the five official working languages of the United Nations. (PDF 763 kb) [file 40249_2017_267_MOESM1_ESM.pdf]

Translation of the abstract into the five official working languages of the United Nations

التأثير النفسي للندوب الناجمة عن داء الليشمانيات الجلدي على طلاب المدارس الثانوية في إقليم الرشيدية، المغرب

عصام بنيس، سيفيرين تيس، هند الفيلالي، فنسنت دي براوفير، حامد صاحبي، مارلين بوليارت

ملخص

خلفية: في المغرب، داء الليشمانيات الجلدي (CL) يعرف عادة بأنه مرض من الأمراض الجلدية الموضعية بطيء الشفاء، ولكن في بعض الحالات، يمكن أن يؤدي إلى ندبات تحدث تشويها. تفشى داء الليشمانيات الجلدي بسبب الليشمانيا الكبيرة في إقليم الرشيدية في جنوب شرق المغرب بين عامي 2008 و2010 وأصاب كثير من المراهقين بندبات دائمة في الوجه أو غيرها من أجزاء الجسم المكشوفة. درسنا التأثير النفسي من لداء الليشمانيات الجلدي على هؤلاء الشباب.

الأسلوب: في عام 2015 أجرينا مسحا مستعرضا بين طلاب المدارس الثانوية الذين يعيشون في مدارس داخلية في منطقتين يتوطن فيهما داء الليشمنيات في الرشيدية: الريصاني وتنجداد. بين استبيان ذاتي تركز حول المعرفة العامة بداء الليشمانيات الجلدي وندباته. ركز سؤال غير محدد الإجابة على الآثار النفسية والاجتماعية المحتملة المرتبطة بهذه الندبات. وقد تم تحليل البيانات الكمية باستخدام Epi Info™ والبيانات النصية باستخدام برنامج NVivo.

النتائج: ذكر حوالي 20٪ من 448 شملهم الاستطلاع انهم عانوا من آفة داء الليشمانيات الجلدي وقال 87٪ أنها قد تؤدي ربما أو بالتأكيد إلى عواقب نفسية. أظهر تحليل النص أن الآثار النفسية على الفتيات كانت أكثر سلبية من الأولاد فيما يتعلق بداء الليشمانيات الجلدي. واعتبر الطلاب داء الليشمانيات الجلدي بأنه "خطير"، و"مخيف"، و "مميت"، وقالوا إنه أدى في بعض الأحيان إلى التفكير في الانتحار.

الاستنتاجات: عبء داء الليشمانيات الجلدي في هذه الفئة العمرية لا يمكن تجاهله. الندوب التي تنتج داء الليشمانيات الجلدي تؤدي إلى ازدراء الذات والشعور بالعار تجاه المجتمع، وظهور الآثار النفسية السلبية في هذه الفئة العمرية. في حين قبل بعض الطلاب ندوب داء الليشمانيات الجلدي وتقبلوها باعتبارها "قدر"، والبعض الآخر يطالب بشدة باتخاذ تدابير وقائية ضد داء الليشمانيات الجلدي وعلاج الندبات.

Translated from English version into Arabic by Mahmoud Sami, through

TRANSLATORS
WITHOUT BORDERS

皮肤利什曼病所致疤痕对摩洛哥 Errachidia 省高中生的社会心理影响

Issam Bennis, Séverine Thys, Hind Filali, Vincent De Brouwere, Hamid Sahibi, Marleen Boelaert

摘要

背景：在摩洛哥，皮肤利什曼病通常被认为是一种可以缓慢自愈的、局部性皮肤疾病。但是，部分病例可引起残疾性疤痕。2008 年-2010 年，摩洛哥东南部 Errachidia 省的皮肤利什曼病暴发导致一些高中生面部或其他肢体暴露部位的永久性疤痕。本研究旨在了解皮肤利什曼病对这些年轻人群的社会心理影响。

方法：2015 年我们针对 Errachidia 省的两个利什曼病流行区（Rissani 和 Tinejdad）的学校的高中生开展了一项横断面调查。通过自我应答式问卷了解学生们关于利什曼病和相关疤痕的一般知识。其中包括一道开放性问题，关于这些疤痕可能引起的社会心理影响。使用 Epi Info 软件分析定量数据，使用 NVivo 软件分析文本数据。

结果：448 名应答者中，约 20%回答曾经有过利什曼病结节，87%回答这些疤痕可能或肯定会引起社会心理问题。文本分析表明女生比男生对皮肤利什曼病更具有负面的社会心理反应。学生们认为皮肤利什曼病是危险的、严重的和致死的，而且有时会引起极端的自杀想法。
结论：皮肤利什曼病对该年龄人群的影响是不可忽视的。无法消除的皮肤利什曼病疤痕导致该年龄人群的自卑和社会歧视以及负面社会心理影响。部分学生接受了皮肤利什曼病所致疤痕及相关的痛楚，认为是自己的命运，而其他学生则急切地需要保护性措施以抗击皮肤利什曼病和处理相关的疤痕。

Translated from English version into Chinese by Men-Bao Qian, through

CDC CHINESE CENTER FOR DISEASE CONTROL AND PREVENTION
NATIONAL INSTITUTE OF PARASITIC DISEASES

Impact psychosocial des cicatrices de la leishmaniose cutanée chez les lycéens de la province d’Errachidia au Maroc

Issam Bennis, Séverine Thys, Hind Filali, Vincent De Brouwere, Hamid Sahibi, Marleen Boelaert

Résumé

Contexte : La leishmaniose cutanée est connue au Maroc comme une maladie de peau localisée qui se résout lentement mais peut laisser des cicatrices mutilantes. L’épidémie de leishmaniose cutanée causée par *Leishmania major* dans la province d’Errachidia, dans le sud-est du Maroc, entre 2008 et 2010, a laissé à de nombreux adolescents des cicatrices indélébiles sur le visage ou d’autres parties du corps visibles. Nous avons étudié l’impact psychosocial de ces lésions sur ces jeunes gens.
Méthodes : Nous avons mené en 2015 une étude transversale parmi des lycéens vivant en internat dans deux régions de leishmaniose endémique dans la province d’Errachidia : Rissani et Tinejdad. Nous avons recueilli leurs connaissances sur la leishmaniose cutanée et les cicatrices qu’elle cause dans un auto-questionnaire. Une question ouverte portait sur les effets psychosociaux possibles de ces cicatrices. Les données quantitatives ont été analysées avec Epi Info™ et les données en texte avec le logiciel NVivo.
Résultats : Près de 20 % des 448 jeunes gens interrogés ont répondu qu’ils avaient eu une lésion de leishmaniose cutanée et 87 % que ces lésions pouvaient « peut-être » ou « certainement » avoir des conséquences psychologiques. L’analyse du texte a montré que les filles détaillaient plus souvent que les garçons les conséquences psychologiques négatives de la leishmaniose cutanée. Les lycéens considéraient la leishmaniose cutanée comme « dangereuse », « grave » et « mortelle », allant jusqu’à écrire qu’elle provoquait parfois des idées suicidaires extrêmes.
Conclusions : Le poids de la leishmaniose cutanée dans ce groupe d’âge n’est pas négligeable. Les cicatrices indélébiles sont négatives pour l’image de soi-même et stigmatisantes socialement, et entraînent des effets psychologiques négatifs dans ce groupe d’âge. Alors que certains lycéens acceptaient leurs cicatrices et considéraient cette souffrance comme « leur destin », d’autres demandaient avec insistance des mesures de protection contre la leishmaniose cutanée et un traitement contre les cicatrices.

Translated from English version into French by Suzanne Assenat, through

Психосоциальное воздействие шрамами из-за кожного лейшманиоза на учащихся средней школы в провинции Эр-рашидия, Марокко

Issam Bennis, Séverine Thys, Hind Filali, Vincent De Brouwere, Hamid Sahibi, Marleen Boelaert

Реферат

Фон: В Марокко, кожный лейшманиоз (КЛ), как правило, медленно исцелении локализованного заболевания кожи, но в некоторых случаях, это может привести к усечению шрамы. Вспышки КЛ за счет *Leishmania major* в провинции Эр-рашидия на юго-востоке Марокко в период между 2008 и 2010 осталось много подростков с рубцовой ткани на лице или других открытых частях тела. Мы изучили социально-психологические воздействия КЛ на этих молодых людей.

Методы: В 2015 году мы провели кросс-секционный опрос среди старшеклассников, проживающих в школах-интернатах в два КЛ-эндемичных районах Эр-рашидии: Rissani и Tinejdad. Самоуправляемой вопросник об общих знаниях КЛ и соответствующие шрамы. Открытый вопрос о возможных психологических эффектах, связанных с этими шрамами. Количественные данные были проанализированы с Epi Info™ и текстовыми данными с программой NVivo.

Результаты: Почти 20% из 448 опрошенных сообщили, что они испытали КЛ очага и 87% сказали, что это возможно или наверняка привести к психологическим последствиям. Текстовый анализ показал, что девочки чаще, чем мальчики расширили негативные психологические последствия КЛ. Ученики рассматривают КЛ как “опасные”, “серьезный”, и “дары”, и сказал, что это иногда приводило к экстремальным суицидальные настроения.

Заключение: Бремя КЛ в этой возрастной группе не является незначительным. Неизгладимый КЛ шрамы привесдёт к самостигматизации и социальной стигматизации, а также возникновению негативных психологических эффектов в этой возрастной группе. В то время как некоторые студенты приняли их КЛ рубцов и их страдания, как их “судьбу”, другие требуют защитных мер против КЛ и лечениЯ шрамов.

Translated from English version into Russian by Hao-Qi, Zhang, through

Impacto psicosocial de las cicatrices producidas por leishmaniasis cutánea en alumnos de escuela secundaria en la provincia de Errachidia, Marruecos

Issam Bennis, Séverine Thys, Hind Filali, Vincent De Brouwere, Hamid Sahibi, Marleen Boelaert

Resumen

Antecedentes: En Marruecos, por lo general se piensa que la leishmaniasis cutánea (LC) es una enfermedad cutánea localizada, de cicatrización lenta, pero en algunos casos puede resultar en cicatrices mutilantes. El brote de LC por *Leishmania major* en la provincia de Errachidia al sudeste de Marruecos entre 2008 y 2010 dejó a muchos adolescentes con tejido cicatricial permanente en la cara y otras partes expuestas del cuerpo. Estudiamos el impacto psicosocial de la LC en estos jóvenes.

Métodos: En el año 2015 se llevó a cabo una encuesta transversal entre alumnos de escuela secundaria que vivían en internados en dos de las áreas endémicas de LC en Errachidia: Rissani y Tinejdad. Un cuestionario autoadministrado obtuvo respuestas sobre conocimiento general de la LC y las cicatrices asociadas. Una pregunta abierta se enfocó en los posibles efectos psicosociales asociados con dichas cicatrices. Se analizó la información cuantitativa con Epi Info™ y la información en forma de texto con el programa NVivo.

Resultados: Casi 20% de los 448 encuestados reportaron que padecieron de lesiones por LC y 87% dijo que posiblemente o definitivamente resultarían en consecuencias psicológicas. El análisis de texto indicó que las mujeres se expandían más que los varones en cuanto a los efectos psicológicos de la LC. Los alumnos consideraron que la LC era “peligrosa”, “seria”, y “mortal”, y que a veces podía derivar en pensamientos extremos de suicidio.

Conclusiones: La carga de la LC en este grupo etario es significativa. Las cicatrices indelebles de la LC resultan en autoestigma y estigma social y en la emergencia de efectos psicosociales negativos en este grupo etario. Si bien algunos de los alumnos aceptaban las cicatrices de la LC diciendo que el sufrimiento era su “destino”, otros ponían empeño en demandar medidas protectoras contra la LC y el tratamiento para las cicatrices.

Translated from English version into Spanish by Maria Alejandra Aguada, through

TRANSLATORS
WITHOUT BORDERS
