# Supplementary material for: Psychosocial impact of scars due to cutaneous leishmaniasis on high school students in Errachidia province, Morocco
Source: Infect Dis Poverty. 2017 Apr 7;6:46. doi: 10.1186/s40249-017-0267-5 (PMC5383955; doi:10.1186/s40249-017-0267-5)
Supplement: Supplementary file 2 — English translation of the self-administered questionnaire. (PDF 327 kb) [file 40249_2017_267_MOESM2_ESM.pdf]

Additional file 2: English translation of the self-administered questionnaire

QUESTIONNAIRE ABOUT CUTANEOUS LEISHMANIASIS (APRIL 2015)

This questionnaire is part of ongoing research about 'Control of Cutaneous Leishmaniasis in Morocco' with the support of the National School of Public Health in Rabat and the Institute of Tropical Medicine in Antwerp, Belgium. The principal aim of this study is to understand knowledge, representations and practices of the population about Cutaneous Leishmaniasis.

Your participation in this questionnaire will help to understand your personal experience with this disease. You will not be asked to give your name and your identity. You can decide at any time to stop answering and withdraw from the study, or not participate. 15 minutes will be enough to complete all the questions by selecting the appropriate answer.

Do you agree to participate in this study:
1. Yes, I agree 2. No, I do not agree

Area: 1.Tinejdad/Ferkla High school 2.My Alicherif /Rissani High school

Actual study level: 1st Year common section 2nd Year 3rd Year

Gender: 1. Man 2. Woman

Age of the participant (in years):

Name of the district where your family home is located:

Do you know a disease named cutaneous leishmaniasis? 1. Yes 2. No

Do you know a local name for this disease, if so, which?

Have you personally experienced this disease?

1. Yes 2. No 3. I do not know

If your last response is "Yes", in which year have you been affected?

Do you know anyone affected by this disease? 1. Yes 2. No

What is your relationship with this/these person(s)?

This disease is transmitted to humans by:
1.Dogs 2.Mosquitoes 3.Water 4.Rodents 5.Garbage 6.Ido not know

Do you think girls are more exposed to get cutaneous leishmaniasis?

1.Yes 2.No 3.I do not know

Does this disease leave marks and scars (cicatrices) in the site of its occurrence?
1.Yes 2.No 3.I do not know

Is it possible to have scars (cicatrices) of this disease on the face?
1.Yes 2.No 3.I do not know

How long to those marks and scars (cicatrices) remain visible in the skin?

1. It becomes less visible in the skin within years
2. It disappears completely
3. It does not disappear and stays visible on the affected skin

Do you think that medical treatment of marks and scars (cicatrices) of Cutaneous Leishmaniasis exist?
1.Yes 2.No 3.I do not know

Is there any traditional remedy that is used to decrease the scars (cicatrices)?
1.Yes 2.No 3.I do not know

If your answer is "Yes" to the above, write which?

In your point of view, why did the number of CL cases in your area decrease in the last years?
(You can select more than one answer)

1. Due to the hygiene campaigns
2. Due to the interventions against the wild rodents (forest)
3. Due to the interventions against the mosquitoes
4. Due to the individual acquired immunity
5. Due to the natural herd immunity of the population
6. Due to the climate change
7. Due to other factors

Do you use, or does your family use at home:

1. Insecticide spray
2.Bednets
3. Smoke or herbs to repel mosquitoes
4. Nothing of the above

During the summer, do you and your family frequently sleep:

1. outside the home or on the terrace
2. inside a room with closed door and windows
3. inside a room with open door and/or windows

Are you afraid for yourself and your family to be affected by cutaneous leishmaniasis in the future?

1. Yes, I am very afraid
2. Yes, I am a little bit afraid
3. No, I am not afraid at all

Is it possible that the marks and scars (cicatrices) of Cutaneous Leishmaniasis influence the psychological state of the person affected?

1. Yes 2. Maybe 3. No influence

Could you write a small paragraph about the probable psychological state of the person (woman or man) affected by those scars?

Thank you very much for finishing this questionnaire
