# Supplementary material for: Psychosocial impact of scars due to cutaneous leishmaniasis on high school students in Errachidia province, Morocco
Source: Infect Dis Poverty. 2017 Apr 7;6:46. doi: 10.1186/s40249-017-0267-5 (PMC5383955; doi:10.1186/s40249-017-0267-5)
Supplement: Supplementary file 3 — Original version of the questionnaire in Arabic. (PDF 1639 kb) [file 40249_2017_267_MOESM3_ESM.pdf]

Additional file 3: Original Questionnaire in Arabic

إستبيان حول مرض الليشمانيا الجلدي أبريل 2015

إن هذا الإستبيان، هو جزء من دراسة في طور الإعداد حول" مكافحة داء الليشمانيا الجلدي في المغرب "برعاية المدرسة الوطنية للصحة العمومية في الرباط ومعهد الطب الاستوائي بأنتويرب . الأهداف الرئيسية لهذه الدراسة هي التعرف على معارف وتصورات وممارسات السكان حول داء الليشمانيا الجلدي .

موافقتك على المشاركة في هذا الاستبيان من شأنه أن يساعد على فهم التجارب الخاصة بك فيما يتعلق بهذا المرض. لن يطلب منك ذكر إسمك أو هويتك. يمكنك أن تقرر في أي وقت وقف الإجابة والإنسحاب، أوعدم المشاركة. 15 دقيقة ستكون كافية لإكمال كل الأجوبة و ذلك بتأطير رقم الجواب المناسب.

**هل أنت موافق على المشاركة في هذا البحث : 1. نعم موافق 2. لا غير موافق**

**المنطقة :** 1. ثانوية تينجداد / فركلة 2. ثانوية مولاي علي الشريف / الريصاني

**المستوى الدراسي :** 1.السنة الأولى 2.السنة الثانية 3.السنة الثالثة

**نوع المشارك :** 1. رجل 2. امرأة

**العمر (بالسنوات) :**

**اسم الجماعة الحضرية أو القروية التي يتواجد بها سكن أسرتك :**

**هل تعرف مرضا يسمى الليشمانيا الجلدية** 1.نعم 2. لا

**في حالة وجود إسم محلي لتسمية هذا المرض, ماهو الإسم المتداول؟**

**هل سبق لك أن أصبت بهذا المرض؟** 1.نعم 2.لا 3.لست أدري
في حالة الاجابة سابقا بنعم في أي عام أصبت به؟

**هل هناك شخص تعرفه أصيب بهذا المرض؟** 1.نعم 2.لا
ماهي درجة قرابتك بهذا الشخص؟

هل ينتقل هذا المرض إلى الإنسان عبر :

1.الكلاب 2.البعوض 3.الماء 4.الفأر 5.الأوساخ 6. لست أدري

هل تظن أن الفتيات أكثر عرضة للإصابة بمرض الليشمانيا الجلدية؟ 1.نعم 2.لا 3.لست أدري

هل يترك هذا المرض ندبات و آثارا (cicatrices) في مكان الإصابة؟ 1.نعم 2. لا 3. لست أدري

هل يمكن أن تظهر ندبات (cicatrices)هذا المرض على مستوى الوجه؟ 1. نعم 2.لا 3. لست أدري

ما هي المدة التي تبقى فيها هذه الندبات و الآثار (cicatrices) ظاهرة في الجلد؟

1. يقل ظهورها على الجلد مع مرور السنين
2. تختفي نهائيا
3. لا تختفي وتبقى ظاهرة بالجلد المصاب

هل تظن أنه يوجد علاج طبي لندبات و آثار(cicatrices)الليشمانيا الجلدية؟ 1.نعم 2.لا 3.لست أدري

هل توجد علاجات تقليدية تستعمل للتقليل من مظهر الندبات(cicatrices)؟ 1.نعم 2.لا 3. لست أدري

في حالة الاجابة بنعم ماهي؟

في رأيك لماذا نقصت الإصابات بهذا المرض في منطقتك خلال السنتين الماضيتين؟ (يمكنك اختيار أكثر من إجابة)

1. نتيجة حملات النظافة
2. نتيجة عمليات القضاء على الفئران الوحشية (الغابوية)
3. نتيجة عمليات محاربة البعوض
4. نتيجة المناعة المكتسبة عند الإنسان
5. نتيجة المناعة الطبيعية للساكنة
6. نتيجة تغير المناخ
7. نتيجة عوامل أخرى

هل تستعمل أنت و أسرتك في المنزل :

1. مبيدات الحشرات
2. شباك مضاد للحشرات (Moustiquaire)
3. الدخان أو الأعشاب لطرد البعوض
4. لا نستعمل أي شيء مما ذكر في الأعلى

في فصل الصيف هل تنام أنت و أسرتك غالبا:

1. خارج غرف المنزل (السطح)
2. داخل غرفة مغلقة النوافذ و الباب
3. داخل غرفة مفتوحة النوافذ أو الباب

هل أنت خائف على نفسك و على عائلتك من الإصابة بمرض الليشمانيا الجلدية في المستقبل؟

1.نعم أنا جد خائف 2.نعم خائف شيئا ما 3. لا لست خائفا على الإطلاق

هل يمكن لمظهر الندبات و آثار (cicatrices) الليشمانيا الجلدية أن تؤثر على الحالة النفسية للمصابة أو المصاب؟ 1. نعم ممكن 2. ربما 3. لا تأثير

ماذا يمكنك أن تكتب في فقرة صغيرة عن الحالة النفسية المحتملة للمصابة أو المصاب بهذه الندبات؟

شكرا جزيلا على اتمامك لهذا الاستبيان
